# Enhancing Cassava Starch Bioplastics with *Vismia guianensis* Alcoholic Extract: Characterization with Potential Applications

**DOI:** 10.3390/polym17030419

**Published:** 2025-02-05

**Authors:** Josiel F. Santos, Crystian Willian C. Silva, Barbara P. G. Silva, Pedro H. Britto-Costa, Cleidilane S. Costa, Larissa Otubo, Artur W. Carbonari, Gabriel A. Cabrera-Pasca

**Affiliations:** 1 Programa de Pós-Graduação em Ciência e Engenharia de Materiais—PPGCEM, Universidade Federal do Pará (UFPA), Ananindeua 67130-660, PA, Brazil; josiel.santos@abaetetuba.ufpa.br; 2 Instituto de Pesquisas Energéticas e Nucleares, Comissão Nacional de Energia Nuclear, IPEN-CNEN/SP, São Paulo 05508-000, SP, Brazil; crystianwill@usp.br (C.W.C.S.); barbara.silva@ipen.br (B.P.G.S.); larissa.otubo@ipen.br (L.O.); 3 Research Center for Gas Innovation, Escola Politécnica, Universidade de São Paulo, São Paulo 05508-030, SP, Brazil; brittopedro@usp.br; 4 Faculdade de Ciências Exatas e Tecnologia, Universidade Federal do Pará (UFPA), Abaetetuba 684440-000, PA, Brazil; cleidilane@ufpa.br

**Keywords:** cassava starch, *Vismia guianensis*, bioplastic, hydroalcoholic extract, plasticizer

## Abstract

This work investigates the incorporation of *Vismia guianensis* alcoholic extract (EAVG) into cassava starch, with the aim of improving its bioplastic properties. Cassava starch was dissolved into distilled water and doped with 0.2%, 0.5%, and 1.0% EAVG under a temperature controlled at the gelatinization point (∼70 °C) and then cast to form bioplastics. The resulting samples were characterized via attenuated total reflectance/Fourier transform infrared spectroscopy (ATR/FTIR), thermogravimetric and differential thermal analysis (TGA-DTA), X-ray diffraction (XRD), scanning electron microscopy/energy dispersive spectroscopy (SEM/EDS), atomic force microscopy (AFM), and mechanical essays, providing insights into chemical composition, thermal stability, crystallinity, surface morphology, and mechanical properties. The results demonstrated that EAVG played an effective role, enhancing the flexibility and stability of the bioplastic with potential use in biomedical applications. Moreover, the results also showed significant improvements in mechanical and thermal properties, suggesting that EAVG is a valuable addition to bioplastics. Therefore, EAVG presents a pathway for advancing bioplastics with enhanced mechanical, thermal, and functional characteristics, with the potential for further advancements in these fields.

## 1. Introduction

The sustainability of the plastics industry plays a crucial role in the future development of various everyday applications, which, in addition to being functional, prioritize low carbon emissions and a circular economy [1,2], in accordance with Sustainable Development Goal 12 (SDG12) and Agenda 2030. Conventional plastics are petrochemical-based polymers that have become a problem for biomes, microbiomes, and the environment, and their accelerated replacement is crucial [3,4,5]. In this context, bioplastics emerge as promising candidates and green alternatives to conventional plastics [1]. However, because of their fragility, several strategies are being explored, such as the addition of reinforcing materials to produce effective composites for a variety of applications. These strategies aim to ensure that the type of reinforcement or the new composite provides an adequate replacement for fossil-based plastics. For example, the use of lignocellulosic fibers, synthetic fibers, clay, carbon nanotubes, and even blends with other polymers has been reported in recent years, creating a variety of environmentally friendly composites [6,7].

These approaches aim to improve the physical, chemical, thermal, and mechanical properties of bioplastics. The resulting reinforced bioplastics are often referred to as “smart plastics” due to the ability of the reinforcement agents to impart additional functional properties, such as electrical conductivity, antimicrobial, antibacterial and antioxidant properties [8,9,10]. These additional characteristics significantly broaden the applications of plastic materials, making them ideal for use in biotechnology and even as metal substitutes, among other possibilities [10,11].

Among bioplastic matrices, starch is one of the most promising biopolymeric matrices for developing biodegradable packaging [12,13]. Starch, which is an abundant polysaccharide in nature, is a semicrystalline granule composed of two main types of glucan polymers: amylopectin and amylose. Depending on the source of the starch, the weight percentage of the amylose and amylopectin components varies from 20–25% to 75–80%, respectively [13]. Generally, starch is obtained from renewable sources, and it presents a relatively low cost and a wide range of applications. Consequently, starch is a highly versatile material that has been used in numerous industries such as food, paper, textiles, chemicals, and pharmaceuticals [12,13]. Given the limitations of native sources, different starch modification techniques are available to improve the processability, mechanical properties, and/or barrier properties of biodegradable films.

In the application of starch for the production of bioplastics and thermoplastics, the addition of plasticizers is mandatory. There is also a search for biobased plasticizers that, when integrated into starch matrices, form a functional product with broad applications [13,14]. Bio-plasticizers are additives derived from biological sources that are added to polymers in order to enhance their mechanical and physical properties. These additives improve the flexibility, softness, texture, and functional properties of plasticized polymers. Common examples of bio-plasticizers include vegetable oils, cardanol, isosorbide esters, and citrates, among others [15]. In addition, plasticizers are generally low-molecular-weight compounds added to polymers to enhance their flowability, facilitate the incorporation of other ingredients, such as fillers, and reduce the temperature required to process bioplastics [13,14,15].

Glycerol, sorbitol, and citric acid, for instance, are commonly used as plasticizers in biopolymers such as starch. These additives aim to increase flexibility by reducing the stiffness of the material’s molecules. Although this can slightly decrease mechanical strength, it allows for easier shaping and overall flexibility. Various compounds, including water, monosaccharides, urea, and formamide, are used as plasticizers due to their ability to disrupt hydrogen bonds and increase elasticity. In addition, compounds with a higher boiling point are preferred as a result of the stability they provide. A notable example is glycerol, a tri-alcohol obtained from natural or petrochemical sources, widely used as a plasticizer to reduce the hygroscopicity of starch bioplastics [16,17]. Another example is polyethylene glycol (PEG), derived from ethylene glycol, which is used as a plasticizer, among many other functions, due to its high solubility in water or other solvents [18,19]. Resins, mainly from trees and plants, are not traditional polymers, as they are not formed by repeating monomer units. Instead, they are complex chemical structures that can be considered precursors to polymer formation [20,21].

Due to its well-known abundance of biodiversity, the Amazon region offers enormous potential for the development of biodegradable materials. A notable example is *Vismia guianensis*, commonly known in the region as “Lacre”. This plant, which can grow up to five meters tall, is endemic to the Amazon rainforest and is a member of the family *Hypericaceae* and the kingdom *Plantae*. It is distinguished by an irregular, multibranched canopy. One of the distinctive characteristics of *Vismia guianensis* is its yellow–orange latex (resin), which can be obtained via incisions in various parts of the trunk of the plant. Traditionally, some Amazonian tribes have used this resin to treat wounds, herpes, and fungal infections on the skin. Motta et al. [22] studied extracts of leaves from *V. guianensis*; [22] evaluated Recently, alcoholic extracts from *Vismia guianensis* leaves (EAVG) have been studied and its anticandida activity was evaluated [22]. Furthermore, the antifungal and the anti-inflammatory activities of the EAVG was showed [23].

In order to understand the properties of *Vismia guianensis*, several studies have focused on investigating its phytochemical characteristics, particularly the alcoholic extract obtained from the bark of this species. The results predominantly indicate the presence of secondary metabolites, such as polyphenolic compounds typical of plant species, including anthraquinones, flavonoids, and tannins [23,24]. Broadly, these polyphenolic compounds are characterized by aromatic rings with hydroxyl groups, organic acids, and acetylated sugars, and they are especially notable for their antioxidant potential, metal ion chelation capacity, and applications in adhesives and paints [25,26]. For example, anthraquinones are widely used as dyes in food, pharmaceuticals, and cosmetics [27]. Tannins, with their ability to form complexes with proteins, gelatins, and alkaloids, are notably applied in metal ion and protein chelation, as well as in the production of fabrics with antibacterial properties [28]. Flavonoids, in turn, due to their significant antioxidant potential, are frequently utilized in cosmetics and medical treatments [29,30].

Given the presence of these components, the interaction between the alcoholic extract of *Vismia guianensis* and cassava starch presents a promising alternative for enhancing the properties of starch-based thin films. This interaction is particularly relevant due to the presence of functional groups, such as hydroxyl and carboxyl, which, when reacting with the amylose and amylopectin chains of the starch in solution, play a crucial role in the establishment of effective intermolecular bonds. These bonds promote the formation of gel network structures, enhancing the flexibility of the films and contributing to the intrinsic functionalities of natural polymers, such as antimicrobial activity, self-adhesiveness, and self-healing properties [29,31,32]. Additionally, these functional groups provide a strategic advantage by enabling the chemical modification of natural polymers, thereby expanding opportunities for the development of new materials and enhancing the characteristics of the resulting composites [32,33,34].

**Figure 1:**
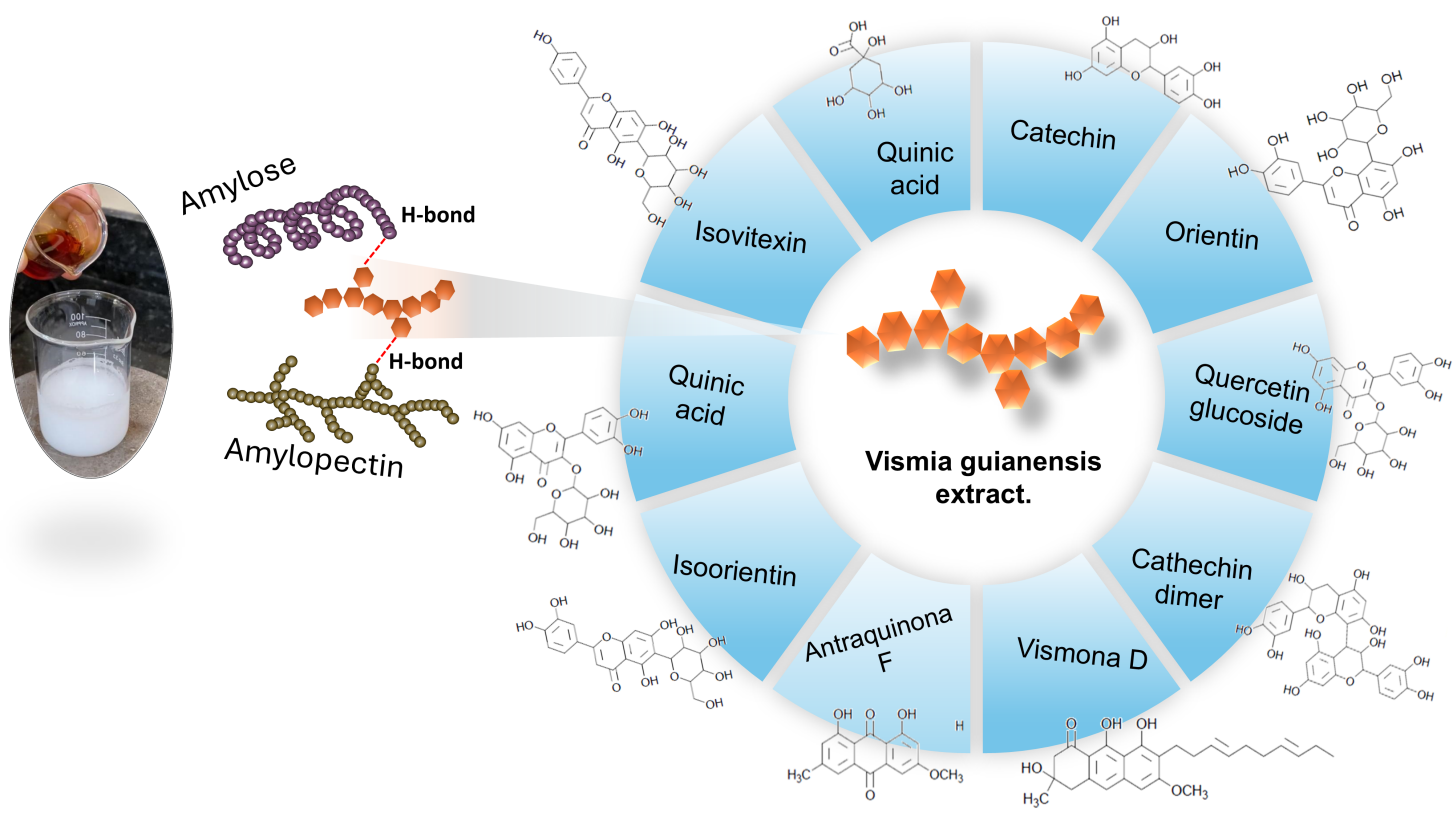
Schematic representation of the metabolites of *Vismia guianensis* extract interacting with amylose and amylopectin from cassava starch, highlighting the hydrogen-bond interactions.

In the work here reported, we present the manufacturing of bioplastics in the form of thin cassava starch films doped with extracts of *Vismia guianensis* sap in isopropyl alcohol (EAVG) from the Abaetetuba region, in the northern state of Pará, Brazil. The properties of EAVG and bioplastics, in the form of a film, were characterized by their microstructural, morphological, chemical, thermal, and mechanical properties. To examine the morphology and microstructure, scanning electron microscopy (SEM) and X-ray diffractometry (XRD) techniques were utilized. The thermal properties were evaluated using thermogravimetric analysis (TGA) and differential thermal analysis (DTA). Concerning the mechanical properties, they were analyzed by stress–strain test curves. For the characterization of EAVG, FTIR, TGA-DTA, and XRD, the analytical techniques were employed. Figure 1 illustrates the synthesis steps and highlights the 14 compounds identified in the alcoholic extract of *Vismia guianensis*. These compounds are capable of interacting with amylose and amylopectin through hydrogen bonding, demonstrating the molecular interactions facilitated via the extracted bioactive compounds.

## 2. Materials and Methods

### 2.1. The Extract of Vismia guianensis in Isopropyl Alcohol (EAVG)

**Figure 2:**
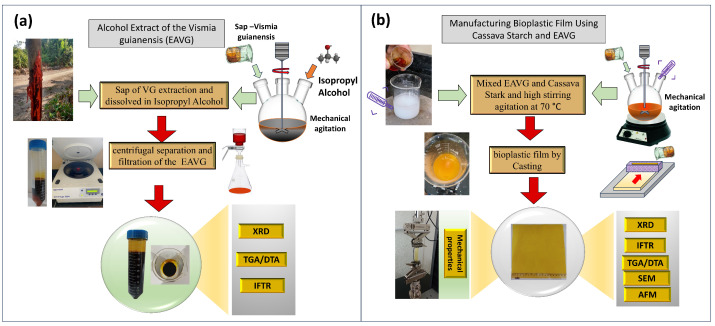
Schemes of (**a**) the experimental methodology for the extraction process: the material was extracted in isopropyl alcohol, centrifuged, and filtered. (**b**) Bioplastic manufacturing methodology: cassava starch was combined with *Vismia guianensis* extract at low concentrations. Physico-chemical characterizations were performed via XRD, TGA/DTA, FTIR, AFM, and mechanical tests.

The extraction of *Vismia guianensis* from tree sap using isopropyl alcohol (EAVG), also known as “Lacre”, was carried out using the following process. Initially, the bark of a VG trunk was carefully stripped away to expose the vessels and access the sap. Eventually, the resin began to flow from the crosscut incisions and was then collected into 50 mL Falcon tubes, using a procedure similar to that of extracting natural latex from rubber trees (see Figure 2a). Isopropyl alcohol was used for the extraction process, and then ultracentrifugation was used to extract EAVG from the sap. This process separated the alcohol-soluble metabolites of interest from the undissolved components and scraps, such as fats, starches, and other organic substances.

In detailed terms, 10 g of sap was mixed with 40 mL of isopropyl alcohol. The mixture was subjected to mechanical stirring (with a stirring speed of 300 rpm) and ultrasound (frequency of 40 kHz) treatment for 30 min. After this step, the solution was transferred to Falcon tubes, followed by centrifugation using an Eppendorf™centrifuge machine at 10,000 rpm for 20 min (this process was repeated three times). Upon centrifugation, undesirable organic compounds precipitated at the bottom of the tube, while the desired portion remained soluble in the alcohol phase. The removal of or reduction in these precipitated compounds is crucial for improving the material’s performance and stability. The resulting solution was filtered through a qualitative filter paper to remove residual particles. This process resulted in the extraction of the “lacre” resin with a concentration of 0.8 g/mL of organic material in isopropyl alcohol, which has a dark orange color (see the illustration in Figure 2a).

The EAVG was characterized through X-ray diffraction (XRD), for which the samples were vacuum-dried for 1 h in a desiccator and measured over an angular range from 5 to 50 degrees. Fourier transform infrared (FTIR) spectroscopy was used to identify functional groups and the chemical composition of the VG extract, with measurements conducted in a dry state. A thermal gravimetric analysis (TGA/DTA) involved placing the dried samples in aluminum crucibles under a synthetic air flow of 50 mL/min at 10 °C/min up to 600 °C to study the thermal stability and decomposition properties of dry EAVG. A schematic drawing of the alcohol extraction process is also depicted in Figure 2a.

### 2.2. Bioplastic Film Manufacturing by Casting Method

Commercial cassava starch from Dona Nuna Ind.e Com.de Derivados da Mandioca LTDA, located in the Brazilian state of Pará, was used to manufacture the biofilms. The process started with the dilution of 15 g of starch in 200 mL of distilled water. This initial solution was mixed in a mechanical stirrer to ensure uniform dispersion.

After mixing, the solution was heated up to the gelatinization temperature of 70 °C, monitored with a digital thermometer. According to Kadam et al. [35], the gelatinization temperature range of cassava starch is 58–70 °C [35]. Once the gelatinization point was reached, a solution of EAVG was added while the temperature was kept at 60 °C. The EAVG concentrations used were 0.2, 0.5, and 1.0 percent with respect to the starch mass. The mixture was then kept at the gelatinization temperature for 30 min to ensure sufficient interaction between the starch and the extract. Bioplastic films were then cast onto a 20 cm × 20 cm laminated white polystyrene substrate. This substrate was chosen because of its “demolding effect”, which prevented the bioplastic films from adhering to its surface. The drying process was carried out under controlled airflow at a temperature of 25 °C to ensure the formation of uniform bioplastic films without bubbles. The films were then ready for subsequent characterization and analysis.

The bioplastic films were characterized using the following techniques: X-ray diffraction (XRD) was used to obtain structural information about the composites. The film samples were mounted on a single-crystal silicon sample holder and scanned on an angular range (2θ) from 5° to 40° using Cu Kα radiation wavelengths. Fourier transform infrared spectroscopy (FTIR) was employed to characterize the chemical interactions between the components of the bioplastic and identify functional groups in the film. Thermal gravimetric analysis/differential thermal analysis (TGA/DTA) was used to assess the thermal stability, decomposition properties, and degradation behavior of bioplastics. Measurements were carried out with a synthetic air flow of 50 mL/min at 10 °C/min up to 600 °C in aluminum crucibles. Atomic force microscopy (AFM) was utilized to investigate surface morphology and grain distribution. Lastly, mechanical properties such as the tensile strength and elasticity of the bioplastics were evaluated. A schematic representation of the alcohol extraction process is depicted in Figure 2b.

## 3. Results

### 3.1. EAVG Characterization

#### 3.1.1. X-Ray Diffraction, TGA-DTA, and SEM-EDS

**Figure 3:**
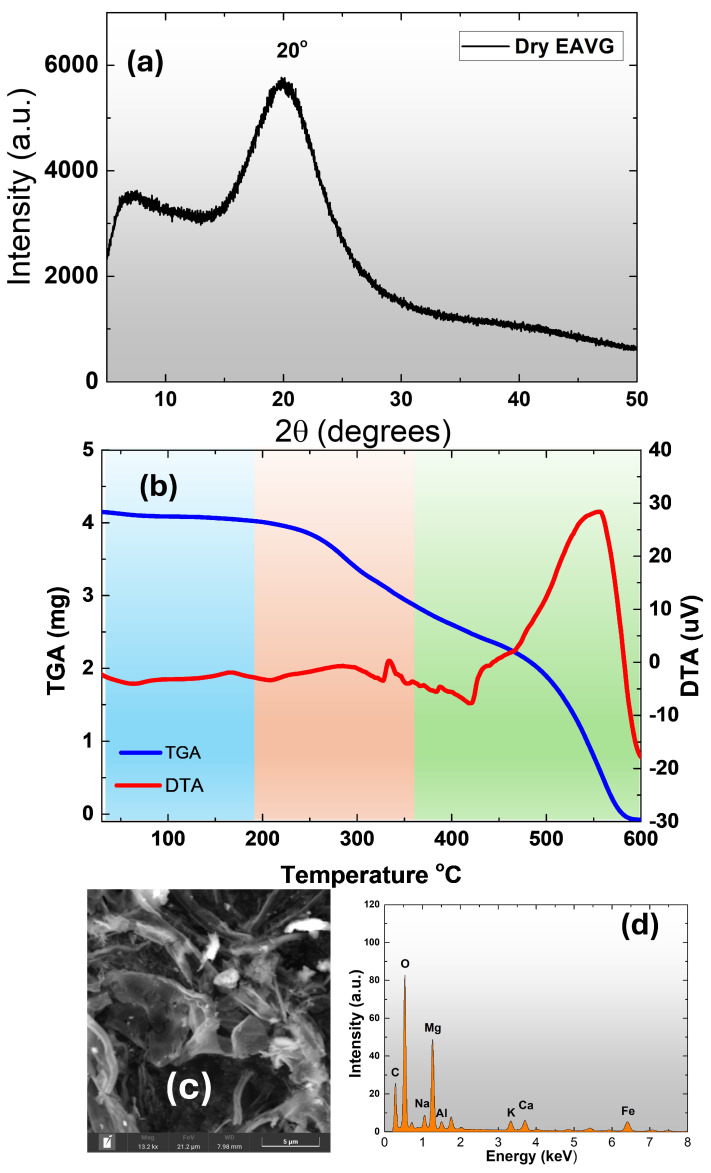
(**a**) X-ray diffraction patterns of EAVG—dry on a monocrystalline silicon substrate. (**b**) TGA-DTA analysis of dry EAVG displaying characteristic thermal behavior. (**c**) SEM image revealing lamellar morphology of residual materials after TGA-DTA measurements. (**d**) EDS analysis identifying natural mineral components (e.g., Na, Mg, Al, Ca, K, and Fe) in the residual materials.

To comprehensively analyze EAVG, its diffraction patterns were obtained by placing dry EAVG on a monocrystalline silicon substrate and measuring 2θ angles ranging from 5° to 50°. The obtained diffraction pattern indicated an amorphous structure, typically due to weak long-range interactions between the dry molecular components of EAVG (see Figure 3a). However, it is important to note that these molecular entities exhibit short-range interactions responsible for the XRD pattern (forming halos around 20°), indicating that the molecular components are not aligned with crystalline planes [36].

To characterize the thermal properties of amorphous EAVG, thermal measurements using TGA-DTA were conducted. The analysis was carried out with a synthetic air flow of 50 mL/min at a heating rate of 10 °C/min in the temperature range from 30 °C to 600 °C, using alumina crucibles. The temperature dependence of TGA (blue curve in Figure 3b) shows a characteristic moisture loss between 30 °C and 200 °C, indicating dehydration, i.e., the loss of mass due to physically adsorbed water molecules (blue region). The orange region (from ∼200 °C to ∼320 °C) corresponds to the mass loss of chemically adsorbed water layers and the depolymerization of EAVG molecules. Subsequently, from 320 °C to 600 °C, there is a mass loss attributed to the degradation of the carbon layers and the formation of CO and CO_2_.

The DTA red curve in Figure 3b shows melting points at 320 °C and 420 °C and an exothermic event around 556 °C due to CO_2_ degradation. After measurements, residual components were analyzed via SEM (scanning electron microscope) images, revealing a lamellar morphology (see Figure 3c). The identification of elements, such as Na, Mg, Al, Ca, K, and Fe, was made possible through EDS inspection, as shown in Figure 3d. This allowed for the identification of natural minerals inherent in the EAVG, similar to minerals found in other plant species [37].

#### 3.1.2. Fourier Transform Infrared Spectroscopy (FTIR)

**Figure 4:**
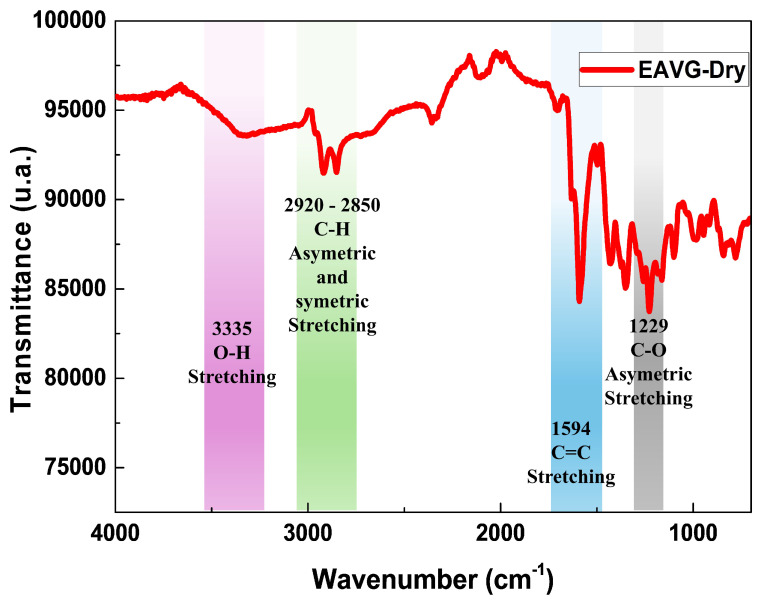
Fourier transform infrared spectroscopy (FTIR) spectrum of EAVG in the frequency range of 4000–600 cm^−1^. The characteristic bands of EAVG-dry, indicated by different colored regions, are highlighted in the spectrum.

FTIR is a powerful analytical technique that can provide important information on the molecular structure of organic molecules and plant extracts, such as the dry ethanol extract of *Vismia guianensis* (EAVG). Figure 4 shows the EAVG–dry infrared spectrum in the frequency range of 4000–700 cm^−1^. In the spectrum, the following characteristic bands of EAVG can be highlighted: the band at 3335 cm^−1^ corresponds to broadband due to O–H stretching vibrations, characteristic of alcohols and phenols. The bands at 2920–2850 cm^−1^ correspond to symmetric and asymmetric C–H stretching vibrations, respectively. The single band at 1594 cm^−1^ corresponds to C=C bonds from aromatic compounds [2]. A strong absorption band at 1229 cm^−1^ is characteristic of the asymmetric stretching of the C–O bond in alcohols, phenols, and carboxylic acids [38].

These bands indicate the presence of flavonoids, amines, and carboxylic acids, which is consistent with the analysis performed by Motta et al. [22]. Using a direct-injection flow of the hydroalcoholic extract of *Vismia guianensis* (EHVG), they identified phenolic compounds such as anthraquinones, catechins, epicatechins, kaempferol, vismione, and flavonoids. The compounds found are the same as those previously described in other studies using leaves of *Vismia guianensis* [39,40].

### 3.2. Bioplastics Characterization

#### 3.2.1. Scanning Electron Microscopy/Energy Dispersive Spectroscopy (SEM/EDS)

**Figure 5:**
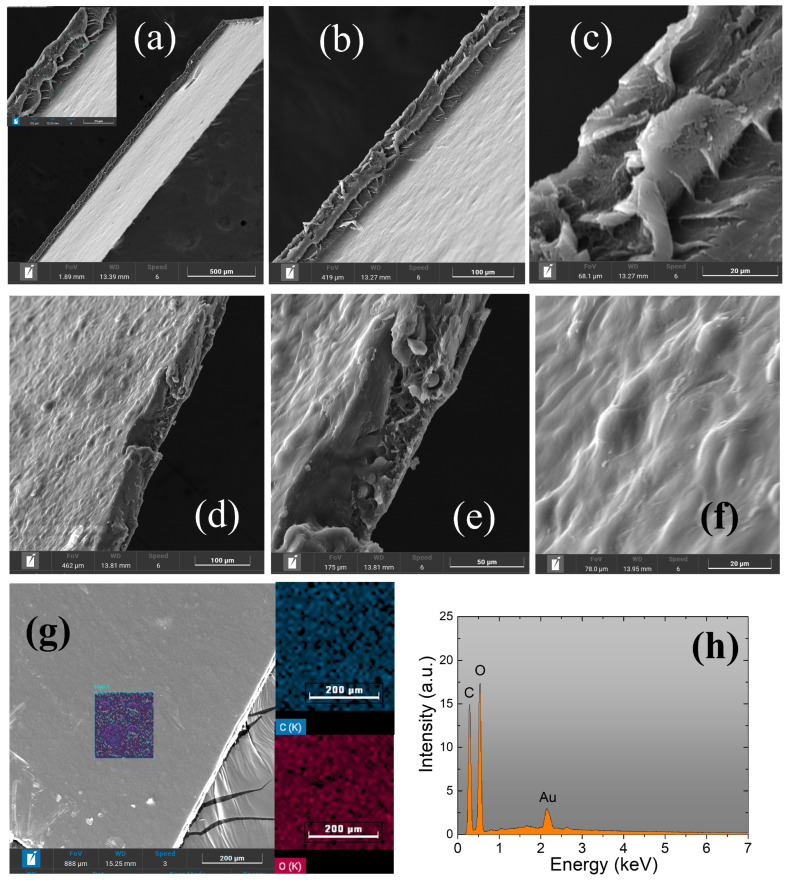
(**a**–**f**) SEM images of the starch sample containing 0.2% EAVG, illustrating the surface morphology and structural details of the biocomposite. EDS images (**g**,**h**) depict the distribution and elemental composition of carbon and oxygen within the sample, providing insights into the integration and interaction of the extract with the starch matrix.

The morphological inspection via SEM/EDS is shown in Figure 5 for starch samples doped with 0.2% EAVG, while the results for samples doped with 0.5% and 1% EAVG are presented in Supplementary Figures S1–S4, respectively, in the Supplementary Materials. Figure 5a corresponds to a cross section of the sample, revealing the internal structure of the bioplastic film. The films showed excellent strength and ease of handling. The film thickness was analyzed along the cross-section in all samples, averaging approximately 70 μm, as shown in Figure 5b. This indicates that the casting process of the bioplastic films allowed precise control of film thickness and uniformity. The image in Figure 5c confirms its uniform thickness and highlights the dense lamellae formation that is typical of all the films produced in this investigation. A fracture in the film is visible in images displayed in Figure 5d,e, exposing characteristic starch particles. A detailed view of the bioplastic film surface, shown in Figure 5f highlights the surface undulations, demonstrating the existing surface roughness of the film. Figure 5g,h show, respectively, the EDS inspection of the surface composition and mapping of the distribution of carbon and oxygen. No other elements were present at detectable levels for the EDS.

#### 3.2.2. Atomic Force Microscopy (AFM): Surface Roughness (SR) and Particle Size (PS)

**Figure 6:**
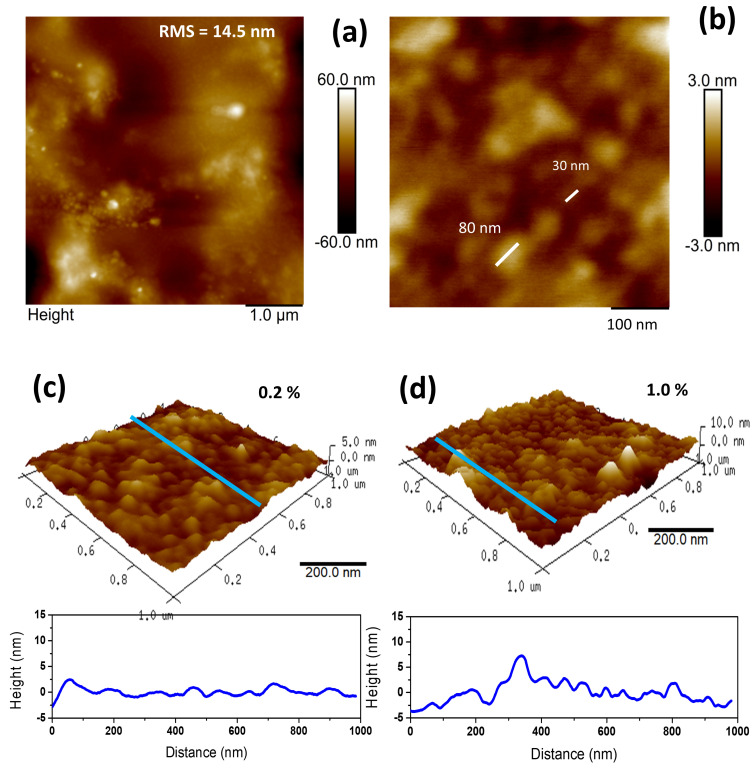
AFM topographic image of 0.2% EAVG bioplastic film with (**a**) 5 μm^2^ and (**b**) 500 nm^2^. Three-dimensional AFM images (1 μm^2^) from (**c**) 0.2% and (**d**) 1.0% of EAVG bioplastic films showing the surface roughness and their respective cross-section analysis.

AFM was utilized to analyze the morphological characteristics of the bioplastic films, including surface roughness and grain size estimation. The presence of nanoparticles was observed on the surfaces of the EAVG bioplastic films (see Figure 6 and Figure S5). These particles have been referred to in the literature as starch nanoparticles (SNPs), and they can be produced through a variety of techniques, including precipitation [41], acid hydrolysis [42], and ultrasonication [43]. The precipitation method involves the use of a nonsolvent, such as alcohol, that should be miscible with the solvent [44].

Isopropyl alcohol was the nonsolvent used in the EAVG extraction process for the EAVG bioplastic film manufacturing. For the 0.2% EAVG sample, a scan size area of 5 μm^2^ was used to measure the roughness (RMS), which was found to be 14.5 nm (Figure 6a). The SNPs were found to have a diameter ranging from around 30 to 80 nm at the 0.2% EAVG film surface (Figure 6b). Figure 6c,d show that a rather smooth surface with nanospheres present was visible in smaller scan sizes.

The cross-section view, which is shown at the bottom of Figure 6c,d, was obtained from the blue line drawn in the 3D images. The RMS was 25.4 nm for the 0.5% EAVG sample and 3.8 nm for the 1% EAVG data (Figure S5). Peak-to-valley values ranged from 0.8 to 2.9 nm for the 0.2% EAVG sample and from 0.9 to 9.5 nm for the 1% EAVG sample. The random SNP distribution in the film matrix may be the cause of this RMS variance [45].

#### 3.2.3. FTIR of Bioplastic Film

**Figure 7:**
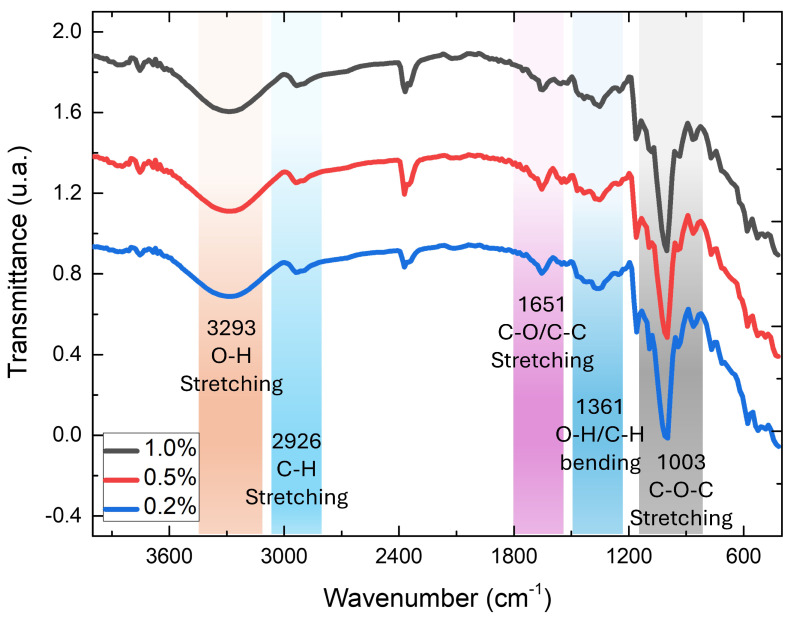
FTIR spectra of bioplastic films with EAVG at concentrations of 1% (black line), 0.5% (red line), and 0.2% (blue line). The characteristic bands, common to all spectra, indicated by different colored regions, are highlighted.

The starch composites with EAVG at different doping levels of 1.0%, 0.2%, and 0.5% were characterized using Fourier transform infrared spectroscopy (FTIR). All spectra shown in Figure 7 display similar bands, specifically the bands corresponding to the pure cassava starch spectra. This was expected since all samples were prepared using a cassava starch matrix with precise concentrations of EAVG and distilled water. As a result, the three spectra exhibited similar characteristic bands [46,47].

The O–H stretching band is observed at 3317.56 cm^−1^, with the broad spectrum indicating extensive hydrogen bonding from both inter- and intramolecular forces [46]. The C–H stretching vibration in the characteristic FTIR spectrum of native starch shows a distinct peak between 2900 cm^−1^ and 2933 cm^−1^. In all obtained spectra, a band around 2925 cm^−1^ is observed, related to the C–H stretching vibrations of aliphatic groups in the hydrocarbon backbone of starch doped with EAVG. The wavenumber 1647.21 cm^−1^ is attributed to a hydroxyl group. According to the literature, the absorption around 1640 cm^−1^ is a typical band present in the spectra of starch and its derivatives, related to strongly bound water [47]. The absorption peaks around 1450–1330 cm^−1^ are associated with the bending and scissoring of CH_2_ (out-of-plane bending) [48]. The characteristic peaks at 1078.5 cm^−1^ and 1003 cm^−1^ for the bioplastic film are related to the C–O stretching bond of starch [46,47,48].

#### 3.2.4. TGA and DTA Thermal Analysis

**Figure 8:**
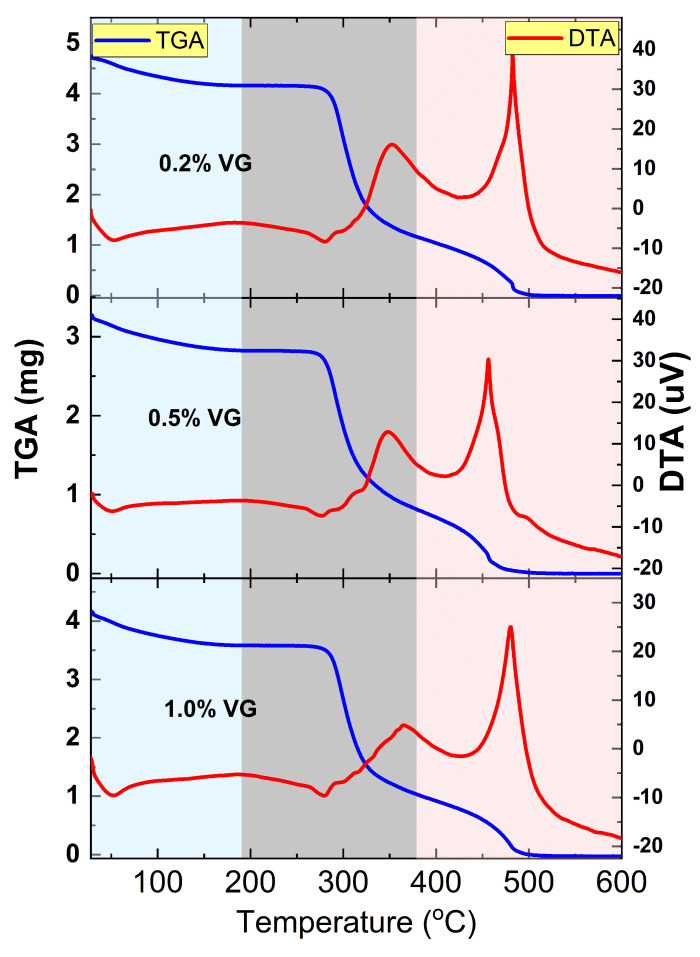
Thermal analyses using thermogravimetry analysis (TGA) and differential thermal analysis (DTA) at the films of cassava starch plasticized with EAVG. The colored regions indicate the observed thermal events.

Thermal analyses recording data from room temperature to 600 °C were conducted using thermogravimetry analysis (TGA) and differential thermal analysis (DTA). The characteristic thermal curves of the material are depicted in Figure 8, and they reveal three distinct thermal events with respect to temperature.

Ranging from room temperature to 200 °C, a decrease of 6% in the TGA curve suggests moisture loss, likely from the residual water used during the preparation of the bioplastic film and environmental interaction. Between 200 °C and 400 °C, a significant mass loss of approximately 65% occurs, peaking at 289 °C as indicated by the derivative in the TGA curve. This region is attributed to the release of physically bound water and volatile carbohydrates, continuing up to 320 °C, beyond which an exothermic peak in the DTA curve at 354 °C indicates the depolymerization of cassava starch molecules forming smaller derivatives like amylose and amylopectin and depolymerization of the EAVG metabolites.

The third stage, which occurs between 400 °C and 600 °C, involves the decomposition of the carbon component of the bioplastic and the degradation of the inorganic oxides formed during thermal analysis. This results in an ash content of approximately 22%. An exothermic peak at 480 °C in the DTA curve suggests energy-releasing polymer oxidation processes. Consistent thermal behavior was observed across all samples (1%, 0.2%, and 0.5% of EAVG) in both TGA and DTA analyses. These findings agree with previous investigations on pure cassava starch [49]. However, experiments involving the introduction of dopants or plasticizers, such as ethylene glycol and other extracts, exhibit different TGA-DTA curves due to varying concentrations and interactions [49,50].

#### 3.2.5. X-Ray Diffraction (XRD) and Stress–Strain Curves Analysis of Cassava Starch Bioplastic Films Doped with EAVG

**Figure 9:**
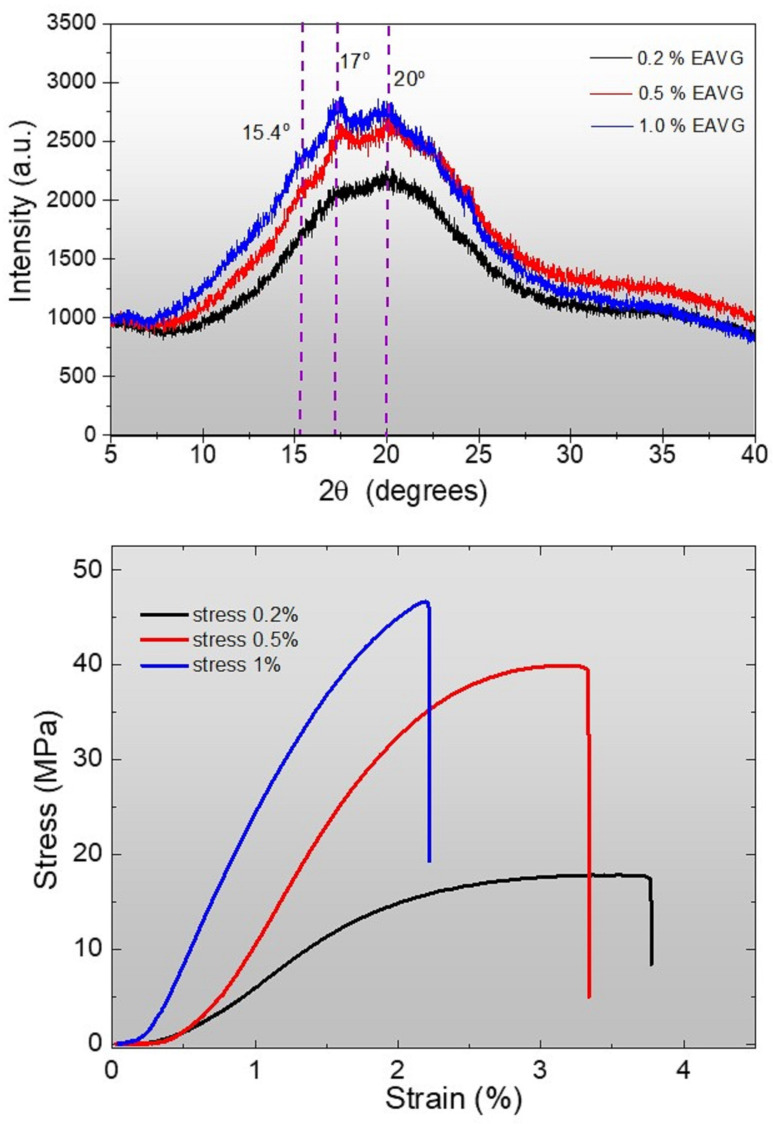
(**a**) X-ray diffraction patterns for bioplastic film with different concentrations of EAVG showing main diffraction peaks at different positions (angles) of 2θ (15.4°, 17°, and 20°, represented by dashed lines in the figure. (**b**) Stress–strain curves of bioplastic films with EAVG, representing the antiplasticization effect.

XRD analysis was performed to examine structural changes in the cassava starch biocomposite matrix when doped with *Vismia guianensis* extract (EAVG). The samples, in the form of films, were placed in a zero-background silicon sample holder on a Rigaku diffractometer. The diffractograms of the cassava starch bioplastic films with 0.2%, 0.5%, and 1.0% EAVG are presented in Figure 9a. Results for the bioplastic films with 0.5% and 1.0% EAVG extract show diffraction peaks at 2θ of 15.2° and 17°, characteristic of the formation of theformation of the $V_{H}$ Helix structure [12,51]. The $V_{H}$-type crystallinity can be transformed into the $V_{A}$-type under dehydration conditions, and vice versa [12,51]. In contrast, the peaks at 17° and 20° tend to disappear, forming a completely amorphous structure in the bioplastic film with 0.2% EAVG. This result indicates that the presence of EAVG prevents starch retrogradation during the cooling process in the casting technique applied to the formation of bioplastic films [52].

The increase in crystallinity has implications for both the tensile strength and the stretchability of the biocomposite films. Therefore, EAVG doping can directly influence the mechanical properties of the biofilms, suggesting that controlling the concentration of EAVG can be an effective strategy to optimize the performance of these materials.

The stress–strain curves demonstrate an antiplasticizing effect of EAVG (Figure 9b), with a decrease in tensile strength and an increase in flexibility as the concentration of EAVG extract decreases. In contrast to the other samples, the bioplastic film containing 1.0% EAVG exhibited high tension and minimal deformation, indicating a higher degree of brittleness. Nevertheless, the bioplastic films with concentrations of 0.5% and 0.2% EAVG exhibited greater ductility, as evidenced by their increased deformation prior to fracture in the mechanical test, further supporting the antiplasticizing effect.

Interestingly, and unlike commonly used plasticizers such as glycerol, sorbitol, and polyethylene glycol (PEG) [53,54], in which an increase in plasticizer concentration typically leads to higher flexibility, our study found that increasing the concentration of EAVG actually resulted in a greater brittleness of the biofilms. The sample with a concentration of 0.2% showed the highest elongation, compared to the sample with 1.0% EAVG, indicating a strong interaction between EAVG and cassava starch molecules (*amylose/amylopectin*) [51].

## 4. Discussion

Since amylose and amylopectin generate strong inter- and intramolecular hydrogen bonds that prevent native cassava starch from functioning as plastics, the frequent use of plasticizing materials is necessary to alter the molecular structure of cassava starch and other types of starch, in order to improve its functionality in various applications. As a result, several chemical and/or physical interactions, including water diffusion, gelatinization, and the polymer fusion of starch granules, are involved in the addition of plasticizers. These interactions help break down the hydrogen bonds and contribute to the formation of a more flexible and moldable starch structure. In the present work, EAVG (an alcoholic extract of *Vismia guianensis*) was added to a solution of cassava starch in distilled water at the gelatinization point, which is around 70 °C. The temperature was measured directly in the solution using a type-K thermocouple. The solution was then cast to form the biofilm, following the methodology described above. The results showed that the bioplastic films had flexible and tensile-resistant properties, which are superior to those reported in the previous literature [13].

It is important to note that the gelatinization point of cassava starch promotes the disintegration of the starch granules, reducing the internal molecular affinity. The polymers found in EAVG, which act as the plasticizing material in this instance, inhibit the formation of inter- and intramolecular hydrogen bonds within the starch granule, thereby partially depolymerizing the structure of cassava starch and creating a flexible structure with an improved dispersion and smoother, lamellar morphology. This is evidenced by SEM images in Figure 5, Figures S1 and S2, in Supplementary Materials for EAVG concentrations of 0.2%, 0.5%, and 1.0%, respectively.

The literature reports that the type and concentration of the plasticizing material influence its efficacy; for example, polar and hydrophilic molecules with a boiling point higher than the temperature required to produce bioplastic films would make an efficient plasticizer for starch. In this regard, EAVG consists essentially of three major groups: flavonoids, ferruginin, and visminones. These groups have the ability to interact with the starch matrix through hydrogen bonding, as they contain hydroxyl (OH) groups, which makes EAVG a platform for the production of bioplastic films and functional plastics.

The characterization results of the bioplastic films in this study reinforce the interaction of metabolites with starch. For example, the X-ray diffraction patterns of all bioplastic films show a predominantly amorphous behavior. It is well known that cassava starch has a low degree of crystallinity (13%), classified as type C, where the characteristic diffraction peaks depend on the type of processing to which it was subjected [13]. The type C crystallinity of cassava starch is formed by the coexistence of A- and B-type crystallites. Specifically, a type C starch granule has a B-type structure nucleus surrounded by A-type crystallites. During the gelatinization process, the crystallinity changes from type C to the $V_{H}$ helix structure, which is unstable and tends to become amorphous [51,52,53,54,55]. This fact was observed in the X-ray patterns of the bioplastic films in this study. In the cases of 1.0% and 0.5%, the occurrence of VH Helix phase peaks was evident; yet, it was not dominant. For the 0.2% concentration, no peaks were observed, showing a characteristic diffraction pattern of an amorphous system. Additionally, AFM data revealed very small grains, in the nanometer range, indicating the action of EAVG in preventing retrogradation during cooling due to the interaction between amylose and amylopectin.

An additional intriguing finding relates to the improved mechanical properties of the 0.2% EAVG bioplastic films. These films performed better under strain, making the bioplastics less brittle and more flexible than pure starch or starch plasticized with urea, which are brittle even at low concentrations [56,57]. Furthermore, the mechanical behavior of the EAVG bioplastic films exhibits resistance to thermal degradation, as they have different degradation points compared to pure starch films and those with plasticizers. Although the difference is small, the increase in the degradation temperature from 0.2% to 1.0% EAVG demonstrates the effectiveness of the EAVG interaction. The results of the mechanical test show lower strain for the 0.5% and 1.0% films compared to the 0.2% EAVG sample. This may be due to the presence of $V_{H}$-type peaks, which indicate phase separation and leaching of the bioplastic film. This can lead to plane slippage and ultimately rupture, resulting in smaller stretches compared to the 0.2% sample, as shown in the stress vs. strain chart in Figure 9b.

It should be noted that Motta et al. [22] found that the alcoholic extracts of *Vismia guianensis* have anti-Candida and anti-inflammatory properties, inhibiting fungal virulence factors associated with the presence of vismione D [22]. This suggests that these extracts may offer a novel treatment approach for diseases caused by Candida [22,39,40]. As such, bioplastic films could be explored as a potential carrier for this type of medication, as well as serving as natural plasticizers for various plastics and thermoplastics that could be developed using this natural extract.

## 5. Conclusions

Several analytical techniques were used to characterize the alcohol extract of *Vismia guianensis* (EAVG) and bioplastic films made of cassava starch doped with EAVG. Fourier transform infrared spectroscopy (ATR/FTIR) was used to identify the functional groups present in the biofilms. Thermogravimetric analysis (TGA/DTA) demonstrated the thermal stability of the biofilms and revealed that, in comparison to pure starch, EAVG delays the degradation process, improving the thermal resistance of the bioplastic films. X-ray diffraction (XRD) analysis confirmed that bioplastic films containing EAVG exhibited predominantly amorphous behavior, with the formation of $V_{H}$ helix-type structures at concentrations of 0.5% and 1.0%, but not at 0.2%, which showed pure amorphous patterns. Scanning electron microscopy (SEM) and atomic force microscopy (AFM) were used to examine the surface topography of the bioplastic films at the nanoscale. The results revealed the presence of very small grains and confirmed that interaction with EAVG prevented starch retrogradation. In terms of mechanical properties, bioplastic films containing 0.2% EAVG exhibited greater flexibility and less brittleness compared to pure starch films or urea-containing films, as reported in previous studies.

The combination of these methods enabled a comprehensive investigation of bioplastic films made from cassava starch and doped with EAVG, revealing enhancements to their structural, mechanical, and thermal properties. The results showed that EAVG functions quite effectively not only as a plasticizer, improving the strength, flexibility, and stability of biofilms, but also has potential advantages in biological applications. These characterization techniques were essential for validating the efficacy of EAVG as a promising additive for the production of biocompatible and sustainable materials.

## Data Availability

The original contributions presented in the study are included in the article/Supplementary Materials, further inquiries can be directed to the corresponding author.

## Supplementary Materials

The following supporting information can be downloaded at: https://www.mdpi.com/article/10.3390/polym17030419/s1, Figure S1: SEM images of 0.5%; Figure S2: EDS images of 0.5%; Figure S3: SEM images of 1.0%; Figure S4: EDS images of 1.0%; Figure S5: AFM images of 0.5% and 1.0%.

## Abbreviations

The following abbreviations are used in this manuscript:

AFM	Atomic force microscopy
ATR	Attenuated total reflectance
DTA	Differential thermal analysis
EDS	Energy dispersive spectroscopy
EAVG	*Vismia guianensis* alcoholic extract
FTIR	Fourier transform infrared spectroscopy
PAC	Perturbed angular correlations
SEM	Scanning electron microscopy
TGA	Thermogravimetric analysis
XRD	X-ray diffraction
