# Supplementary material for: Enhancing Cassava Starch Bioplastics with Vismia guianensis Alcoholic Extract: Characterization with Potential Applications
Source: Polymers (Basel). 2025 Feb 5;17(3):419. doi: 10.3390/polym17030419 (PMC11819721; doi:10.3390/polym17030419)
Supplement: Supplementary file 1 [file polymers-17-00419-s001.zip › polymers-3310829-supplementary.pdf]

Supporting Information

Enhancing Cassava Starch Bioplastics with *Vismia guianensis* Alcoholic Extract: Characterization with Potential Applications

Josiel F. Santos [1], Crystian Willian C. Silva [2], Barbara P. G. Silva [2], Pedro H. Britto-Costa [3], Cleidilane S. Costa [4], Larissa Otubo [2], ArturW. Carbonari [2,*] and Gabriel A. Cabrera-Pasca [3,4,*]

1 Programa de Pos-Graduacao em Ciencia e Engenharia de Materiais—PPGCEM, Universidade Federal do Para (UFPA), Ananindeua 67130-660, PA, Brazil
2 Instituto de Pesquisas Energeticas e Nucleares, Comissao Nacional de Energia Nuclear, IPEN-CNEN/SP, Sao Paulo 05508-000, SP, Brazil
3 Research Center for Gas Innovation, Escola Politecnica, Universidade de Sao Paulo, Sao Paulo 05508-030, SP, Brazil
4 Faculdade de Ciencias Exatas e Tecnologia, Universidade Federal do Para (UFPA), Abaetetuba 684440-000, PA, Brazil
* Correspondence: carbonar@ipen.br (A.W.C.); gpasca@ufpa.br (G.A.C.-P.)

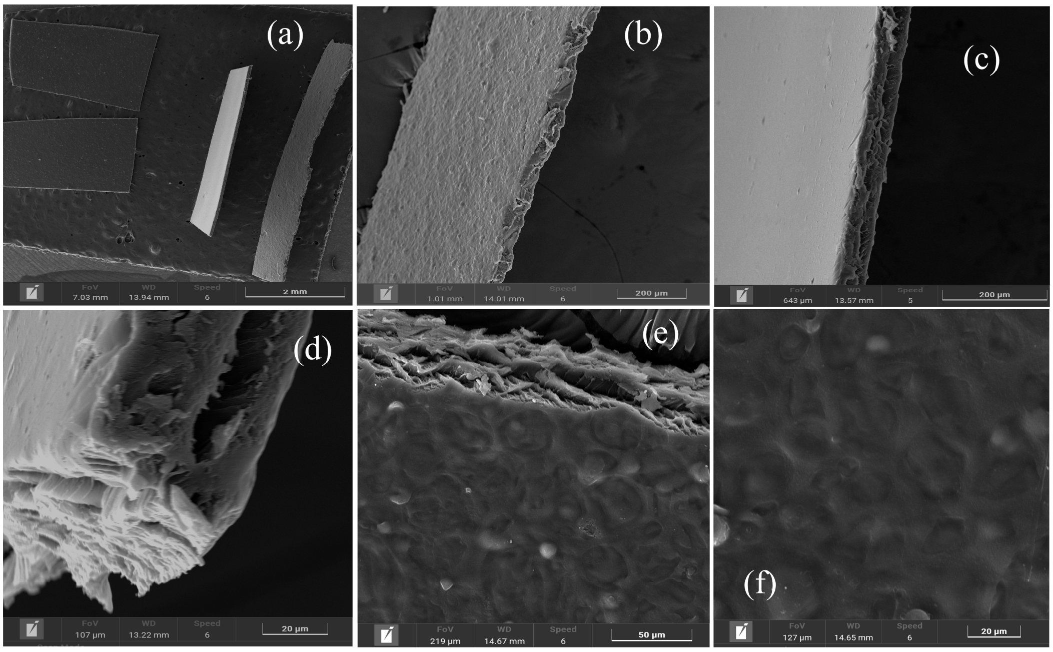

Figure S1. SEM images corresponding to the starch sample containing 0.5% of *Vismia guianensis* alcoholic extract in different configurations and magnifications. (a) Samples mounted on stubs for SEM characterization. (b-d) Cross-section. (e-f) Film surface.

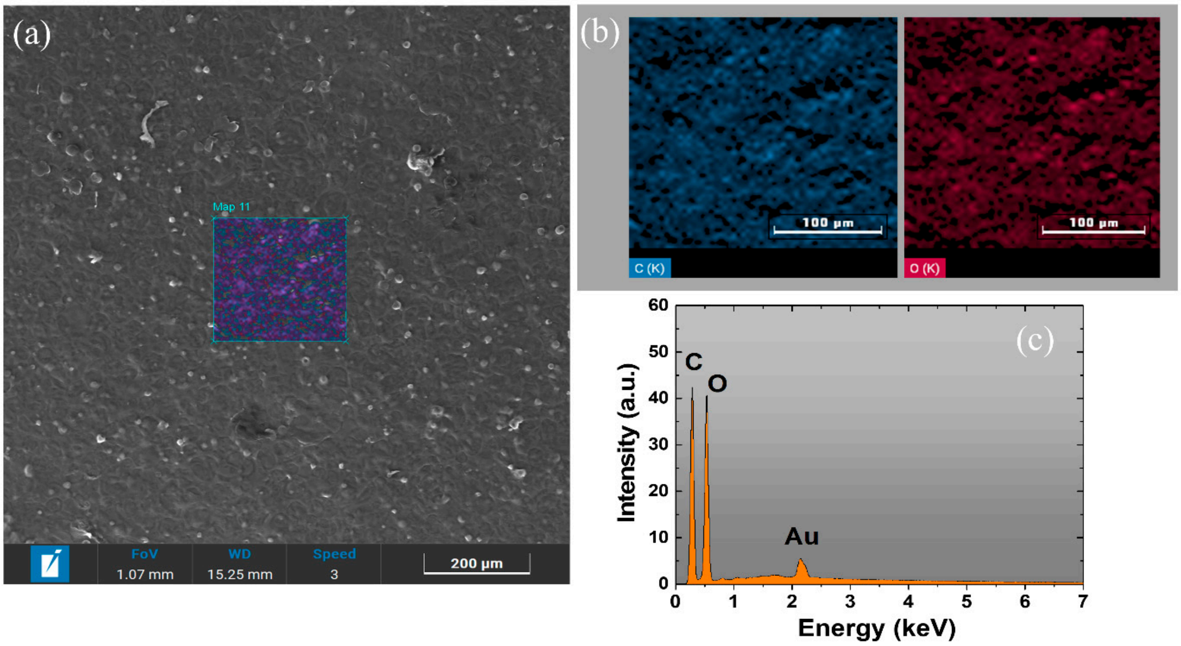

Figure S2. Microscopic and elemental characterization of the starch sample containing 0.5% of *Vismia guianensis* alcoholic extract. (a) SEM-EDS micrograph in Mapping mode. (b) Composition maps. (c) EDS spectrum.

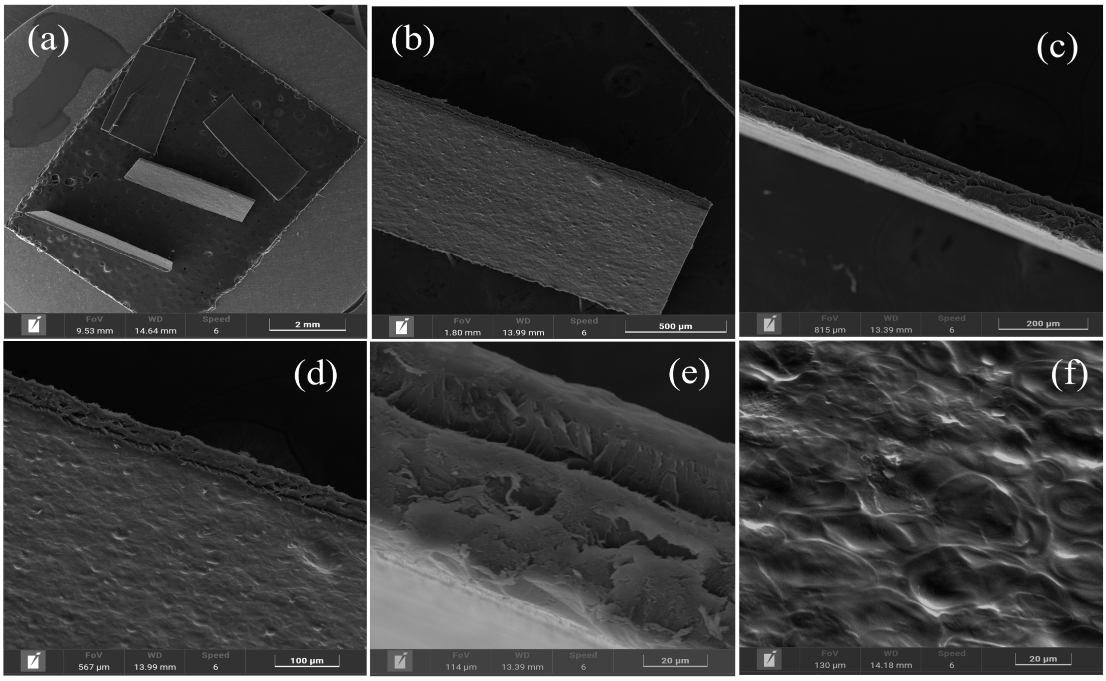

Figure S3. SEM images corresponding to the starch sample containing 1% of *Vismia guianensis* alcoholic extract in different configurations and magnifications. (a) Samples mounted on stubs for SEM characterization. (b-e) Cross-section. (f) Film surface.

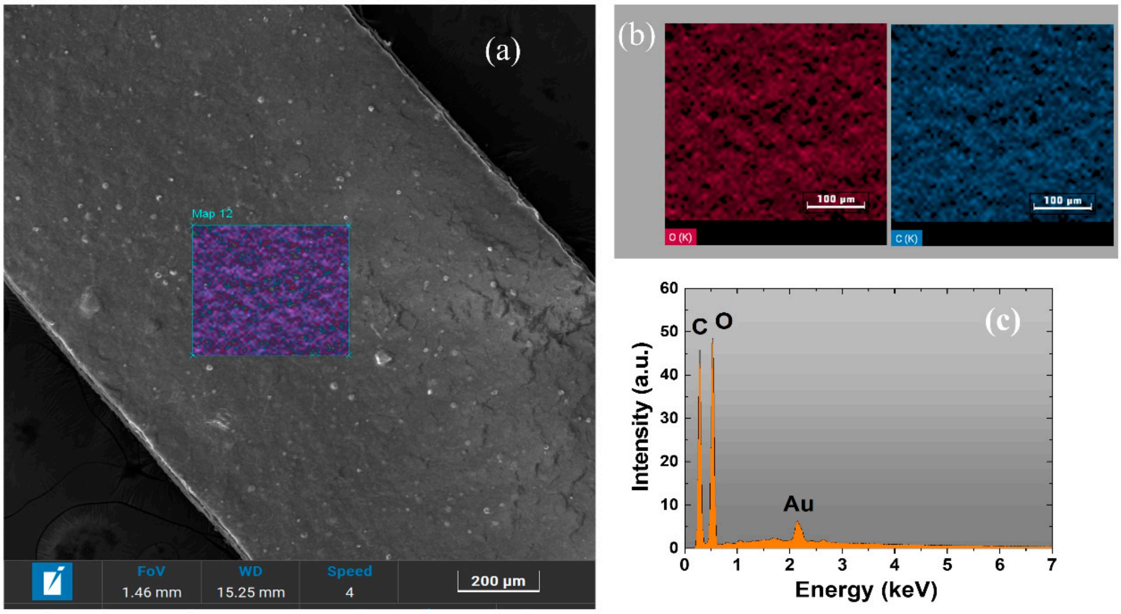

Figure S4. Microscopic and elemental characterization of the starch sample containing 1% of *Vismia guianensis* alcoholic extract. (a) SEM-EDS micrograph in Mapping mode. (b) Composition maps. (c) EDS spectrum.

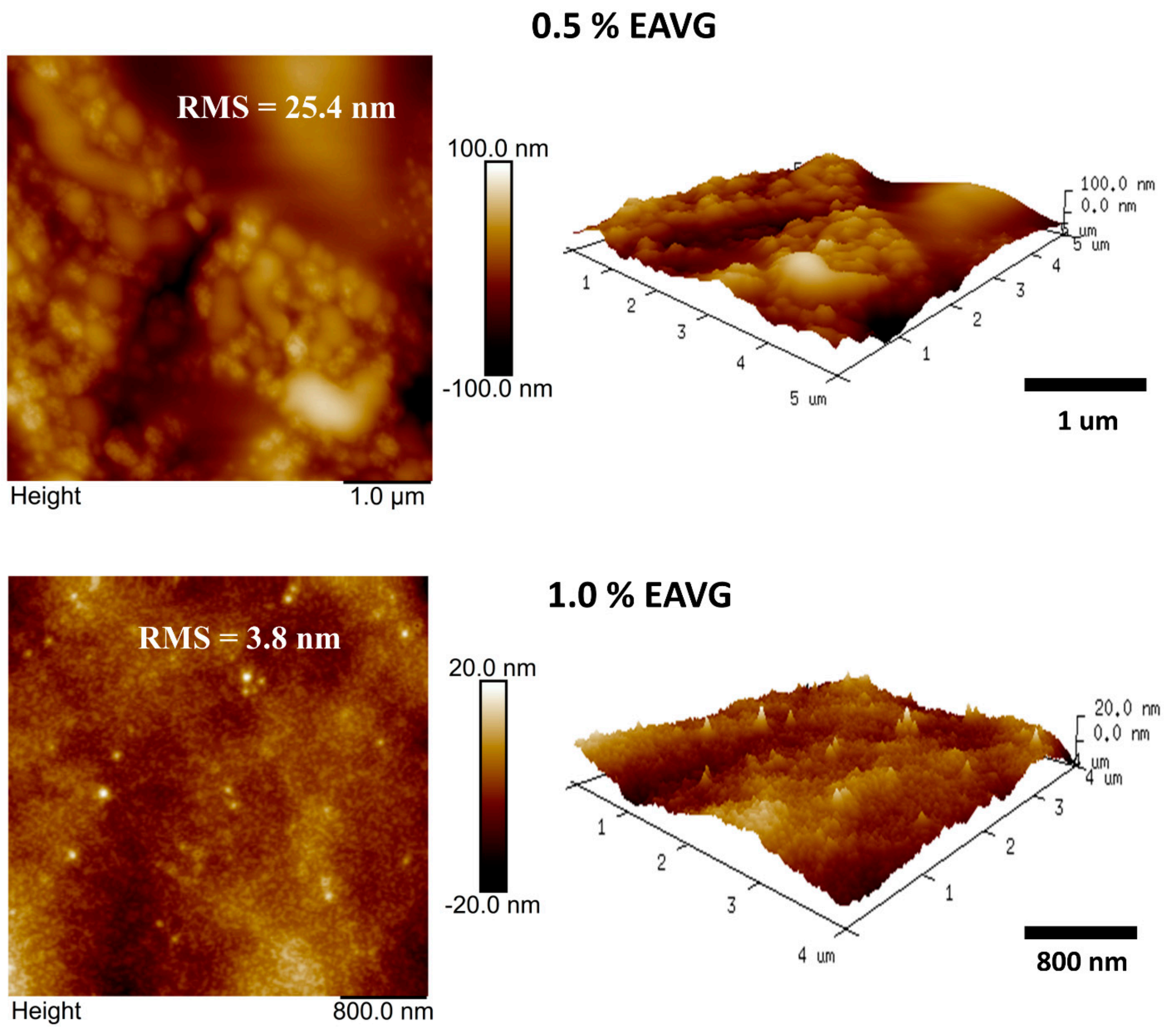

Figure S5. 3D AFM images for 0.5% (top) and 1.0% (bottom) of *Vismia guianensis* alcoholic extract bioplastic films showing the surface roughness and their respective cross section analysis.
